# Salivary Diagnostics: A Brief Review 

**DOI:** 10.1155/2014/158786

**Published:** 2014-01-29

**Authors:** Narasimhan Malathi, Sabesan Mythili, Hannah R. Vasanthi

**Affiliations:** ^1^Department of Oral Pathology & Microbiology, Faculty of Dental Sciences, Sri Ramachandra University, No. 1, Ramachandra Nagar, Porur, Chennai, Tamil Nadu 600116, India; ^2^Department of Biotechnology, Pondicherry University, Puducherry 605014, India

## Abstract

Early detection of disease plays a crucial role for treatment planning and prognosis. Saliva has great potential as a diagnostic fluid and offers advantage over serum and other biological fluids by an economic and noninvasive collection method for monitoring of systemic health and disease progression. The plethora of components in this fluid can act as biomarkers for diagnosis of various systemic and local diseases. In this review paper, we have emphasized the role of salivary biomarkers as diagnostic tools.

## 1. Introduction

Human diseases having global impact include cancer, cardiovascular, metabolic, and neurological diseases. Diagnosing these disease conditions is becoming challenging and thus requires supplementing clinical evaluation with laboratory testing [1]. Salivary diagnostics holds great promise as an effective modality for the early diagnosis, prognostication, and monitoring posttherapy status. Whole saliva is a mixture of the secretions of the major and minor salivary glands, mucosal transudations, gingival crevicular fluid, serum and blood derivatives from oral wounds, desquamated epithelial cells, expectorated bronchial and nasal secretions, bacteria and bacterial products, viruses and fungi,other cellular components, and food debris. It is a complex fluid containing an entire library of hormones, proteins, enzymes, antibodies, antimicrobial constituents, and cytokines [2]. The mechanism of entry of these constituents from the blood into the saliva is by transcellular, passive intracellular diffusion and active transport, or paracellular routes by extracellular ultrafiltration within the salivary glands or through the gingival crevice [3, 4]. The many advantages of saliva as a clinical tool over serum and tissues are noninvasive collection of sample, smaller sample aliquots, good cooperation with patients, cost effectiveness, easy storage and transportation, greater sensitivity, and correlation with levels in blood. Promising new technologies have unveiled large numbers of medically valuable salivary biomarkers for different disease conditions including cancer, autoimmune, viral, bacterial, cardiovascular, and metabolic diseases [2].

## 2. Potential Biomarkers in Saliva

**Figure 1:**
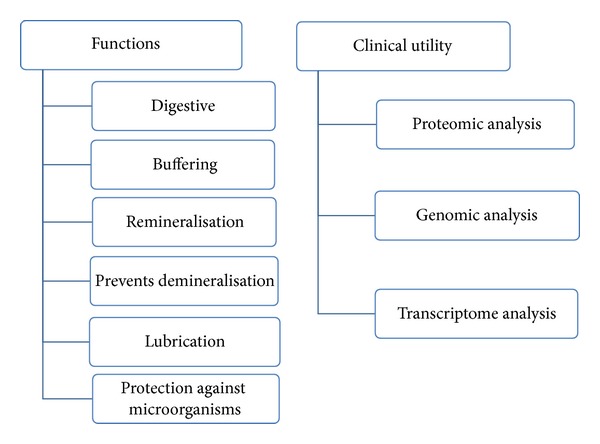
Functions and clinical utility of saliva.

**Figure 2:**
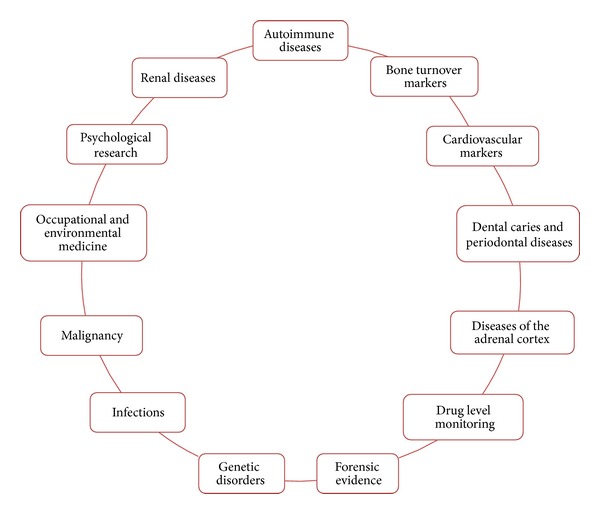
Salivary diagnostics in various systemic diseases.

The wide spectrum of molecules present in saliva provides valuable information for clinical diagnostic applications (Figure 1). Whole saliva is most frequently used for diagnosis of systemic diseases, because it can be easily collected and it contains most of the serum constituents. Salivary diagnostics can be used for the following diseases/conditions (Figure 2) [4].

### 2.1. Autoimmune Diseases

#### 2.1.1. Sjogren's Syndrome (SS)

It is an autoimmune disorder characterized by reduced secretion of the salivary glands and lacrimal glands and associated endocrine disturbance. Sialochemistry offers great value in the diagnosis of SS. An increase in the levels of immunoglobulins, inflammatory mediators, albumin, sodium, and chloride and a decrease in the level of phosphate are indicative of SS. Salivary protein analysis demonstrated an increased level of lactoferrin, beta 2 microglobulin, lysozyme C, and cystatin C. However, the levels of salivary amylase and carbonic anhydrase were decreased [5, 6].

#### 2.1.2. Multiple Sclerosis

Multiple sclerosis (MS) is an inflammatory disease characterized by loss of myelin and scarring caused due to destruction/failure of myelin producing cells by the immune system. Salivary diagnostics shows no significant change in the saliva of patients with multiple sclerosis except for a reduction in IgA production [7].

#### 2.1.3. Sarcoidosis

Sarcoidosis is an inflammatory disease of the lymph nodes, lungs, liver, eyes, skin, or other tissues. Salivary diagnostics demonstrates a decrease in the secretion volume of saliva in addition to a reduction in the enzyme activity of alpha-amylase and kallikrein in most of these patients. However, there was no correlation between the decrease in the enzyme activity and the secretion volume [8].

### 2.2. Bone Turnover Markers

Saliva can be used in mass screening for metabolic bone disorder. Human saliva was analysed for deoxypyridinium (D-PYR) and osteocalcin (OC). Significant correlations have been reported between age, body mass index, D-PYR, or OC concentration and calcaneus T scores. This suggests that saliva could be used as a fluid for assay of human biomarkers of bone turnover. Scannapieco et al. noted a positive association between alveolar bone loss and salivary concentrations of hepatocyte growth factor and interleukin-1 beta. However, there was a negative association between alveolar bone loss and salivary osteonectin. The increased levels of alkaline phosphatase (ALP) activity in periodontitis have been correlated with the alveolar bone loss [9, 10].

### 2.3. Cardiovascular Diseases

Acute coronary syndromes (ACS) refer to a group of clinical syndromes which includes ST-elevation myocardial infarction, non-ST-elevation myocardial infarction, and unstable angina. It is characterized by atherosclerotic plaques which rupture and cause clinical symptoms ranging from chest pain to acute myocardial infarction (AMI). Endothelial injury is the important key event that initiates the atherosclerotic process and inflammation goes hand in hand with this process. Salivary markers of cardiovascular diseases include C-reactive protein (CRP), myoglobin (MYO), creatinine kinase myocardial band (CK-MB), cardiac troponins (cTn), and myeloperoxidase, which, when used in combination with an ECG, shows a positive correlation with myocardial infarct patients as compared to healthy controls. The salivary MYO levels are significantly higher within 48 h of onset of chest pain in AMI patients. Furthermore, salivary MYO levels are correlated positively with its serum concentrations. Although CK-MB and troponins are detected in the saliva, they have poor diagnostic capability [11]. In a study performed by Miller et al., they found that the salivary concentrations of CRP, TNF-*α*, and MMP-9 were significantly higher in patients with AMI and the salivary concentrations correlated positively with the serum concentrations. In addition, salivary myeloperoxidase levels were also found to be elevated in AMI patients. Studies have revealed that salivary soluble ICAM-1 is significantly elevated in AMI patients whereas salivary soluble CD40 ligand is significantly lower in AMI patients [12]. Increased levels of salivary lysozyme have been shown to be associated with hypertension, an early stage of cardiovascular disorders [13, 14].

### 2.4. Dental Caries and Periodontal Diseases

Saliva also has its use in monitoring the level of oral bacteria in it. The increased numbers of *Streptococcus mutans* and lactobacilli in saliva have been associated with increased caries prevalence and root caries [4]. Periodontal diseases have been associated with increased levels of aspartate aminotransferase (AST) and alkaline phosphatase (ALP). Salivary AST can be used as a marker for monitoring periodontal disease. Lower levels of uric acid and albumin in the saliva were associated with periodontitis and diabetes. This could be attributed to the oxidative stress present in the oral cavity during these conditions [15]. In patients with type 2 diabetes mellitus, the salivary expression of pIgR, Arp 3, CA VI, and IL-1Ra was downregulated, whereas PLS-2, LEI, and IGJ chain appeared to be upregulated [16].

### 2.5. Diseases of the Adrenal Cortex

The diseases of the adrenal cortex can be divided as hyperfunction and hypofunction of the gland. The hyperfunctional adrenocortical syndromes include primary hyperaldosteronism, Cushing's syndrome, and adrenogenital syndrome. The hypofunctional syndromes include Addison's disease and selective hypoaldosteronism.

Measurement of elevated late-night salivary cortisol usually at 2300 to 2400 hours is a very reliable tool to the diagnosis of Cushing's syndrome. However, salivary cortisol measurements for diagnosis of adrenal insufficiency have not yet been established [17].

### 2.6. Drug Level Monitoring

Saliva has gained importance for its use for drug monitoring and detection of illicit drugs. Saliva is used to detect the presence of nicotine, cannabinoids, cocaine, phencyclidine, opioids, barbiturates, diazepines, amphetamines, and ethanol. In drug level monitoring, only the unbound fraction of the drug in serum diffuses into the saliva and is detectable in the saliva. The most important diagnostic application of saliva is in the evaluation of illicit drug use. The drug appears in the saliva during the same duration as the serum and thus its mere presence is satisfactory for forensic purposes [18, 19]. Salivary nicotine levels can be used to monitor exposure to tobacco smoke. The major metabolite of nicotine, cotinine, present in the saliva was found to be indicative of active and passive smoking [20]. Rapid detection of illicit drug use can also be done through the direct analysis of methamphetamine, cocaine, and 3,4-methylenedioxymethamphetamine in saliva by a hydrophobic porous silicon array [21]. The endogenous nature of the *γ*-hydroxybutyric acid (GHB) in the blood and urine has posed problems for the forensic toxicologist during drug abuse. Saliva is a biological matrix used for drug testing of GHB levels because of its merit of easy, noninvasive collection and stability of the drug. Also, the values of the drug in saliva correlate with its level in the blood [22].

### 2.7. Forensics

Salivary analysis has been widely used for forensics. The salivary samples can be easily obtained from glasses, cigarettes, food products, envelopes, and other sources. A wide majority of patients secrete blood group antigens into their saliva which can be used for the identification of crime suspects and for paternity law suits [23]. DNA is relatively stable in the dry state; thus DNA testing can be done from the salivary samples. Identification of DNA in saliva by genetic profiling can be helpful in cases of sexual abuse and harassment. The foreign DNA tends to be present in the victim's saliva for as long as 60 minutes providing a valuable piece of forensic evidence [24].

### 2.8. Genetic Disorders

#### 2.8.1. Cystic Fibrosis

Cystic fibrosis (CF) is a genetically determined condition which is caused due to a mutation in the CFTR gene. Saliva is modified in CF patients. The CFTR protein is expressed in the epithelial cells of the parotid gland causing parotid gland involvement. The level of activity of cathepsin-D in saliva of CF patients is significantly higher than in healthy controls before the stimulation of excretion with paraffin pledgets [25].

The values of sodium, potassium, and chloride concentrations were significantly higher than healthy subjects, thus making saliva a diagnostic tool for CF. Salivary calcium concentration, magnesium concentration, and lactate dehydrogenase levels were increased in CF patients when compared with healthy controls [26].

#### 2.8.2. Ectodermal Dysplasia

The most common form of ectodermal dysplasia is the X-linked hypohidrotic ectodermal dysplasia (HED). Lexner et al. performed a study on whole saliva flow and composition in males affected by HED and the female carriers. He found that there was reduced whole saliva flow and the concentration of inorganic constituents and total protein was high. However, the activity and the concentration of the alpha-amylase in the saliva were reduced [27].

### 2.9. Infections

Diagnosis of bacterial and viral pathogens in the saliva is based on combination assay that measures both antibody and antigen or antibody and nucleic acid.

#### 2.9.1. Viral Infections

The key advantage of the diagnostic tests for viruses and bacteria is the identification of a single target. The oral mucosal transudate (OMT) which is obtained by swabbing the buccal mucosa and tongue contains a mixture of sIgA, IgG, IgM, and a rich source of antibodies [4].

In a study performed by Oliveira et al., the measles virus-specific IgM was detected in the saliva. Thus, salivary IgM detection forms a suitable noninvasive method for routine clinical use [28].

Diagnosis of HIV (human immunodeficiency virus) which causes AIDS is possible by antibody-based screening assays. The confirmatory test is a reactive antibody assay which is either a Western blot test via blood or saliva or a polymerase chain reaction via blood. These tests detect p24 antigens and antibodies against both HIV-1 and HIV-2. Also, the salivary secretions contain a variety of salivary proteins which has effective anti-infective activity. However, the detection of viral RNA becomes difficult owing to the decreased viral load [29].

The diagnosis of hepatitis virus first lies in the detection of the antibodies. The confirmatory test for the hepatitis virus infection is a Western (immunoblot) assay combined with a nucleic acid-based viral load assay. Although several such tests are available, none have been proven to be effective with a saliva sample. Salivary diagnostic tests have been designed for detection of human papilloma virus by polymerase chain reaction [30].

#### 2.9.2. Bacterial Infections

Detection of *Mycobacterium tuberculosis* in the saliva is by Polymerase chain reaction during the acute phase of the disease when the bacterial load is high [4, 31]. *Helicobacter pylori* (*H. pylori*) is Gram-negative, microaerophilic bacterium which plays an important role in the natural stomach ecology. It is found in patients with chronic gastritis and gastric ulcers. *H. pylori* binds to salivary mucins MUC-5B and MUC 7 secreted by the mucous and serous acinar cells of the seromucous salivary glands, respectively. Higher levels of salivary MUC-5B and MUC 7 could be used as an indicator for infection with *H. pylori* [32, 33].

#### 2.9.3. Fungal Infections

Salivary diagnostics can also be used for the detection of oral fungi. The salivary fungal count analysis provides valuable information in cases of oral candidiasis. The alterations in the salivary proteins, like immunoglobulins, Hsp70, calprotectin, histatins, mucins, basic proline rich proteins and peroxidases also have important diagnostic value in these cases [34, 35].

### 2.10. Malignancy

Early detection is the key to good prognosis in almost all types of cancer. Saliva has been used as a diagnostic medium for oral squamous cell carcinoma (OSCC), and salivary analytes such as proteins, mRNA, and DNA have been used in their diagnosis.

Aberrant expressions of long noncoding RNA (lncRNA) are associated with lung, breast, and prostate carcinomas. In their study, Tang et al. found that saliva contained a detectable amount of lncRNA which could be potential markers of OSCC [36].

Dysregulation of miRNAs (short noncoding RNA molecules) can be associated with many diseases. miRNAs were found present in the saliva of OSCC patients which could be used as an adjunctive tool to its diagnosis [37].

Salivary mRNA biomarkers (CCNI, EGFR, FGF19, FRS2, and GREB1) can aid in the noninvasive and economical diagnosis of lung cancer [38]. The salivary mRNA biomarkers for detection of ovarian cancer are AGPAT1, B2M, BASP2, IER3, and IL1B [39].

*p53* is a tumor suppressor protein produced in cells in response to various types of DNA damage in the cells. Inactivation of the p53 protein during mutation is one of the leading causes of human cancer development. The p53 antibodies can be detected in the sera and saliva of patients diagnosed with OSCC [40].

*CA15-3* is a tumor marker which is found on the surface of cancer cells and sheds into the blood stream. This tumor marker was found in the saliva of women diagnosed with breast cancer. It is used to monitor advanced and metastatic cases [41, 42].

*c-erb-2* is a receptor tyrosine kinase. Approximately 25% of primary breast and ovarian tumors were found to overexpress the protein. Elevated levels of this tumor marker were found in saliva of patients diagnosed with breast cancer when compared with patients with benign lesions and healthy controls [42].

*CA 125* is a tumor associated antigen which is found to be elevated in the serum and saliva of patients with oral, breast, and ovarian tumors [43].

Fibroblast growth factor 2 (FGF2) and fibroblast growth factor receptor 1 (FGFR1) concentrations in saliva are significantly elevated in patients with salivary gland tumors making it a potential biomarker in the early detection of salivary gland tumors [44].

Prostate specific antigen (PSA) is an established marker of prostate adenocarcinoma (PA). Salivary PSA levels correlate with serum PSA levels in patients with PA. This can serve as a useful biomarker of PA [45].

Salivary cortisol levels were found to be significantly increased in the plasma and saliva of patients of OSCC. This suggests that this hormone can be used as a marker for clinical staging [46]. Salivary lactate dehydrogenase levels are higher in OSCC which makes it a future marker. Elevated levels of salivary nitrate and nitrite were found in patients with oral cancer [47].

Salivary adenosine deaminase (ADA) activity is significantly increased in squamous cell carcinoma of the tongue progressively from stage I to stage III. This biomarker could aid in the early diagnosis of SCC of the tongue [48].

### 2.11. Occupational and Environmental Medicine

Salivary biomarkers play a role in the diagnosis of occupational stress and heavy metal toxin poisoning. Salivary biomarkers associated with occupational stress are classified into two types. Chronic stress is associated with increased levels of salivary cortisol and decreased level of salivary IgA and lysozyme. Saliva chromogranin (Cg)A and alpha-amylase are markers of acute stress [49]. Occupational toxins such as lead and cadmium can also be analyzed from the saliva. The concentration of cadmium in saliva is higher than in blood [50]. But the level of salivary lead in analysis is limited to higher levels of lead exposure poisoning [51].

### 2.12. Psychological Research

Subjects with stress and pain are monitored for changes in the salivary biomarkers since drawing of blood from these subjects can induce more stress and pain. The salivary biomarkers identified are salivary amylase, substance P, secretory IgA, cortisol, and lysozyme. There is an increase in the level of salivary amylase and a decrease in the secretory IgA levels in conditions of psychological stress [5]. Increased levels of salivary immune defense protein chaperone Hsp 70 have also been noted [52]. Salivary testosterone levels have been correlated with the aggressive behavior and athletic activities [53].

### 2.13. Renal Diseases

The various salivary markers associated with end stage renal disease included nitrite, pH, sodium, chloride, uric acid, cortisol, alpha-amylase, and lactoferrin [54]. Salivary phosphate has been widely used as a clinical biomarker for hyperphosphatemia. These levels have correlated well with serum creatinine and glomerular filtration rate [55]. Thus salivary phosphate may serve as a superior marker than serum phosphate levels for diagnosis of heart disease and chronic renal failure [56].

## 3. Conclusion

Saliva has been viewed as an important diagnostic fluid for a very long time now. In recent times, because of the improved efficiency of genomic and proteomic technologies, the use of salivary diagnostics in a clinical setting is becoming a reality. Salivary metabolomics is a new advancement in the field of salivary diagnostics which analyzes a large array of low molecular weight endogenous metabolites present in the saliva for the detection of diseases. Another major improvement is the development of the Oral Fluidic NanoSensor Test (OFNASET) by the UCLA Collaborative Oral Fluid Diagnostic Research Center for the real-time, ultrasensitive, and ultraspecific detection of multiple salivary protein and nucleic acid targets in disease conditions. The greatest milestone in salivary diagnostics is to identify the disease biomarkers and to transfer it from the laboratory to the clinical practice. But the growth of salivary diagnostics has been hindered because of lack of sensitive detection methods, lack of correlation between the biomolecules in the blood and saliva, and the circadian variations in saliva. However, unlike blood and other body fluids, salivary diagnostics offers an easy, inexpensive, painless, and stress free approach to disease detection (Table 1).

## Figures and Tables

**Table 1:** Summary of the salivary biomarkers in various systemic diseases.

S. number	Diseases	Biomarkers	Source of biomarkers
(1)	Autoimmune diseases (1) Sjogren's syndrome (2) Multiple sclerosis (3) Sarcoidosis	Lactoferrin, beta 2 microglobulin, lysozyme C, cystatin C, salivary amylase, and carbonic anhydrase IgA production Alpha-amylase and kallikrein	Saliva

(2)	Bone turnover markers	Body mass index, D-PYR, OC concentration, calcaneus T scores, hepatocyte growth factor, interleukin-1 beta, salivary osteonectin, and ALP activity	Serum and saliva

(3)	Cardiovascular markers	Cardiac troponins, C-reactive protein, myoglobin, myeloperoxidase, ICAM-1, CD 40, and salivary lysozyme	Serum and saliva

(4)	Dental caries and periodontal diseases	*Streptococcus mutans* and lactobacilli count, aspartate aminotransferase, alkaline phosphatase, uric acid, albumin, pIgR, Arp 3, CA VI, IL-1Ra, PLS-2, LEI, and IGJ	Saliva

(5)	Diseases of the adrenal cortex	Salivary cortisol	

(6)	Drug level monitoring	Nicotine, cannabinoids, cocaine, phencyclidine, opioids, barbiturates, diazepines, amphetamines, ethanol, cotinine, methamphetamine, endogenous *γ*-hydroxybutyric acid, and 3,4-methylenedioxymethamphetamine	Serum and saliva

(7)	Forensic evidence	Blood group antigens and DNA testing	Saliva

(8)	Genetic disorders (1) Cystic fibrosis (2) Ectodermal dysplasia	Cathepsin-D, sodium, potassium, chloride, calcium, magnesium, and lactate dehydrogenase Inorganic constituents, total protein	Saliva

(9)	Infections (1) Viral infections (2) Bacterial infections (3) Fungal infections	Measles virus-specific IgM HIV—HIV-1, HIV-2—antibodies, salivary proteins *Mycobacterium tuberculosis*, MUC 5B, and MUC 7 Candidiasis immunoglobulins, Hsp 70, and calprotectin, histatins, mucins, basic proline rich proteins, and peroxidases	Serum and saliva

(10)	Malignancy	lnc RNA, miRNA, CCNI, EGFR, FGF19, FRS2 and GREB1, AGPAT1, B2M, BASP2, IER3, and IL1B, p53, CA15-3, C-erb2, CA 125, FGF 2, PSA, cortisol, lactate dehydrogenase, silver nitrate and nitrite, and salivary adenosine deaminase	Serum and saliva

(11)	Occupational and environmental medicine	Salivary cortisol, IgA, lysozyme, chromogranin, alpha-amylase, lead, and cadmium	Serum and saliva

(12)	Psychological research	Salivary amylase, cortisol, substance P, lysozyme, secretory IgG, and testosterone	Saliva

(13)	Renal diseases	Cortisol, nitrite, uric acid, sodium chloride, pH, alpha-amylase, and lactoferrin. Salivary phosphate, serum creatinine, and glomerular filtration rate	Serum and saliva
